# Inventory of Mental Health Services in Academia and Researchers’ Awareness of Their Availability: Mixed Method Research Protocol and Pilot Study in Switzerland

**DOI:** 10.3389/ijph.2025.1607982

**Published:** 2025-05-21

**Authors:** May Thet Nu Noe, Anaïs Masserey, Anita Bober, Stefan T. Mol, Irina Guseva Canu

**Affiliations:** ^1^ Centre for Primary Care and Public Health (Unisanté), University of Lausanne, Lausanne, Switzerland; ^2^ Amsterdam Business School, University of Amsterdam, Amsterdam, Netherlands

**Keywords:** mental health, occupational health services, higher education institutions, mixed method, pilot study

## Abstract

**Objective:**

To inventory occupational health services (OHS) in European higher education institutions (HEI) and assess researchers’ awareness of these services.

**Methods:**

The protocol, validated in Switzerland, combined a mapping study of OHS with semi-structured interviews with researchers. Data were analyzed using MAXQDA software and triangulated with OHS inventory data.

**Results:**

OHS in 14 Swiss HEI typically include basic medical consultations, mental health counselling, and legal advice. Access varies by users’ status, often favoring students. Service varies across institutions, creating potential inequalities. At one Swiss HEI, twelve researchers were interviewed; stress derived primarily from time pressure and work overload. Respondents desired better communication with management and a more supportive environment. Despite the availability of four OHS at this HEI, awareness of, and confidence in these services were low, and confidentiality concerns led many to seek external support.

**Conclusion:**

Interviewees had limited awareness of available mental health related OHS, and most decided not to rely on institutional HEI services due to confidentiality related concerns. External psychological help services appear more trusted and potentially more effective.

## Introduction

Higher education institutions (HEI) have a responsibility to ensure the occupational health and safety for their employees (i.e., researchers (including PhD and post-doctoral fellows), teachers, technical and administrative staff). This includes providing occupational health services (OHS) that support both physical and mental wellbeing [1]. According to the World Health Organization (WHO), an optimal and healthy workplace involves active collaboration to continually improve health and safety [2], reflecting a holistic view of employees’ wellbeing beyond just the absence of illness [3]. Recent literature highlights an increased prevalence of mental health problems in academia, particularly burnout, depression, anxiety, and stress. Evidence from several studies reports that one-third of the academic community experiences these problems [4–11]. Although mental health trends in academia may mirror those in the general population, particularly among young people, surveys from the US and Europe show that rates of stress, fatigue, and burnout nearly doubled during the COVID-19 pandemic compared to pre-pandemic levels, with a disproportionate impact on women and younger academics [4–7]. Researchers frequently report dissatisfaction with working conditions and a high intention to leave their position [12, 13]. This contributes to reduced commitment and increased turnover, further raising concerns about mental health in academia [14, 15]. The factors contributing to mental health problems include heavy workload, insufficient resources [16], imbalance between efforts and reward [17, 18], and low decision latitude [19]. Although some HEI have implemented interventions, few were adequately evaluated and communicated [20].

To address this gap, the EU COST Action Researchers’ Mental Health Observatory (https://projects.tib.eu/remo/) developed a harmonized protocol to assess the availability, accessibility, and effectiveness of OHS in HEIs. This protocol includes a comprehensive inventory of existing OHS services and aims to facilitate comparison across institutions. A pilot study was conducted in Switzerland that has several top-ranked universities and a legal obligation to ensure occupational safety [21]. The aim of the study was to assess the feasibility of the protocol, which we now present along with its findings. The protocol is designed to help institutions identify and address gaps in occupational health provision, supporting the mental and physical wellbeing of academic staff and facilitating replication in other countries.

### General Requirements for a Harmonized Research Protocol

A harmonized protocol should enable the investigators to apply similar inclusion and exclusion criteria, identical interview guides and questionnaires, and standardized operational procedures (e.g., participant recruitment, interview conduct, data collection, and processing). This standardization allows for data analysis and synthesis both at the HEI and country levels, as well as when pooled together [22]. Mixed-method research is defined by combining a primary or core technique with one or more methods from a different approach to address the research issue through data collection or analysis [23]. To ensure the generalizability of study findings at national and HEI levels, the representativeness of both HEIs and researcher’s samples is important and achievable either via random selection or exhaustive inclusion of the HEI and researcher. However, the former is more manageable, especially when resources are limited.

## Methods

### Study Protocol

For this protocol, we adopted a sequential design, in which the mapping study and review of OHS per HEI preceded the study based on individual semi-structured interviews with OHS users in different HEIs. This sequence facilitated triangulation between the OHS inventory and users’ responses by combining qualitative and quantitative methods.

The original protocol was approved by the Ethical Committee of the University of Lausanne (CER-FBM: 112022-00012C). The protocol and the guide for the semi-structured interviews are available through the Unisanté data repository at https://doi.org/10.16909/dataset/50. Hereafter, we describe its main components and their articulation.

### Mapping Study (Stage 1)

The mapping study consisted of the identification and description of OHS based on a systematic data search and review. It aimed at an exhaustive mapping of OHS available in the HEIs considered. Out of 42 HEIs existing in Switzerland, we sampled all cantonal universities to ensure representation across all geographic regions and types of HEIs, considering factors such as size, seniority, and scientific orientation of the institution.

To identify available OHS, we reviewed the institutional websites and interviewed representatives of the different OHS. For each HEI we documented the following information: name of services; their aim and type of help provided; personnel involved; access details (e.g., how to arrange an appointment, location, opening hours, prices, and usage/application/registration conditions). The information collected was analyzed using the mapping study method [24–26]. The resulting inventory of OHS was presented as an MS Excel database stratified per HEI. Each HEI was then contacted to cross-check and validate their data.

Data were systematically coded and subjected to descriptive analysis. The variables employed in this study were meticulously constructed to capture essential attributes of HEIs and their associated support services. HEI names and types were categorized into universities or (hybrid) research institutions, while geographical categorization was based on canton. Quantitative measures such as the number of students, PhD candidates, and research staff were gathered to characterize institutional demographics.

For the analysis of OHS, a comprehensive set of variables was defined, encompassing the type of service (including occupational medicine consultation, psychological counselling service, juridical/legal counselling, professional orientation counselling, social affairs service, administrative support, and spiritual counselling), establishment year, responsible personnel, geographical location, and financial coverage policies. Financial charges were categorized as: 1- All consultations covered by the institution (free for the user) but sometimes in a limited number (e.g., less than 3 or 5 consultations), 2- Only the 1st consultation is free, subsequent sessions are charged at a reduced rate, 3- Only the 1st consultation is free, subsequent sessions are charged at the normal rate (without discount), and Not provided (NP). Additionally, types of assistance provided (such as consultation/advice, diagnosis, therapy, and administrative help) and the target audience (all, pre-graduated and PhD students, and employees such as researchers, professors, and administrative staff) were documented.

Statistical analyses involved univariate and multivariate linear and logistic regression models to evaluate the relationship between the number of students, PhD candidates, and research staff and the provision of occupational health services. Statistical analyses were performed using Stata software, version 18.

### Qualitative Study Based on Individual Interviews With Researchers (Stage 2)

The aim of this stage is to assess for each HEI, the state of researchers’ (dis)stress and wellbeing at work, their perceptions regarding the protective and risk factors with respect to their mental health, and their knowledge of OHS available at their HEI to preserve and promote their mental health. While the latter corresponds to the study outcomes (i.e., enabling estimation of knowledge completeness, OHS accessibility, and usefulness), the former provided the contextual information that would later be compared and complemented with data from the REMO-STAIRCASE survey (STAIRCASE: https://projects.tib.eu/staircase/).

Semi-structured face-to-face interviews, conducted by a trained clinical psychologist (AB), were used to collect data from individual researchers. The interviews were guided by a list of topics, subsequently developed into themes, and standardized for a duration between 40–60 min. For the quantitative assessment of awareness level, we used the OHS inventory.

The study sample consisted of academic researchers (i.e., PhD students, post-doctoral researchers, research assistants, lecturers, scientists, and professors) currently employed at a Swiss HEI. Exclusion criteria were i) not having research experience at the HEI in question, ii) inability to communicate in English, or iii) refusal to participate (absence of individual informed consent). We employed a random selection process, stratified by the academic position/degree within the HEI, and contract type (permanent versus fixed term). The researchers were contacted using the internal full employee list (intranet) of the investigated HEI, ensuring each researcher had an equal probability of being selected.

The selected researchers received an email from the study PI (IGC) containing information regarding the study and participation modalities. The psychologist’s contact information was provided to enable voluntary participants to schedule the interview directly, ensuring confidentiality. In case of an insufficient participation rate, snowball sampling was employed to complement this process, particularly to reach individuals who may have experienced mental health concerns at work. This option was introduced during the interview by the study psychologist and the participant can relay the information to his/her eligible colleagues to encourage their participation.

Prior to the interview, participants were informed that they could refuse to answer any interview question that might involve a certain level of discomfort, ability, or embarrassment. Their (dis)agreement with the recording of the interview was also noted. In case of refusal, the psychologist took notes and made a complete electronic interview transcript directly after the interview.

All transcripts were de-identified and processed using MaxQda software for content and thematic analysis [27]. The latter facilitated the import and organization of interviews, allowing for structured categorization by location, topic, and research interest. It also enabled precise coding of interview data, enhancing the accuracy and thoroughness of our analysis. The thematic analysis was performed by two independent researchers with background in clinical psychology (AB and AM) according to a rigorous process, following the six-phase approach by Braun and Clark (2006). This approach serves as a sound guide for researchers to understand the phases of thematic analysis and conduct their research more deliberately and systematically [28].

### Data Analyses and Triangulation

Quantitative analysis focused on OHS data from Stage 1, while qualitative thematic analysis from Stage 2 explored researchers’ experiences. Findings from both stages were integrated through triangulation.

## Results

### Mapping Study and Inventory of Occupational Health Service in Swiss HEIs

**FIGURE 1:**
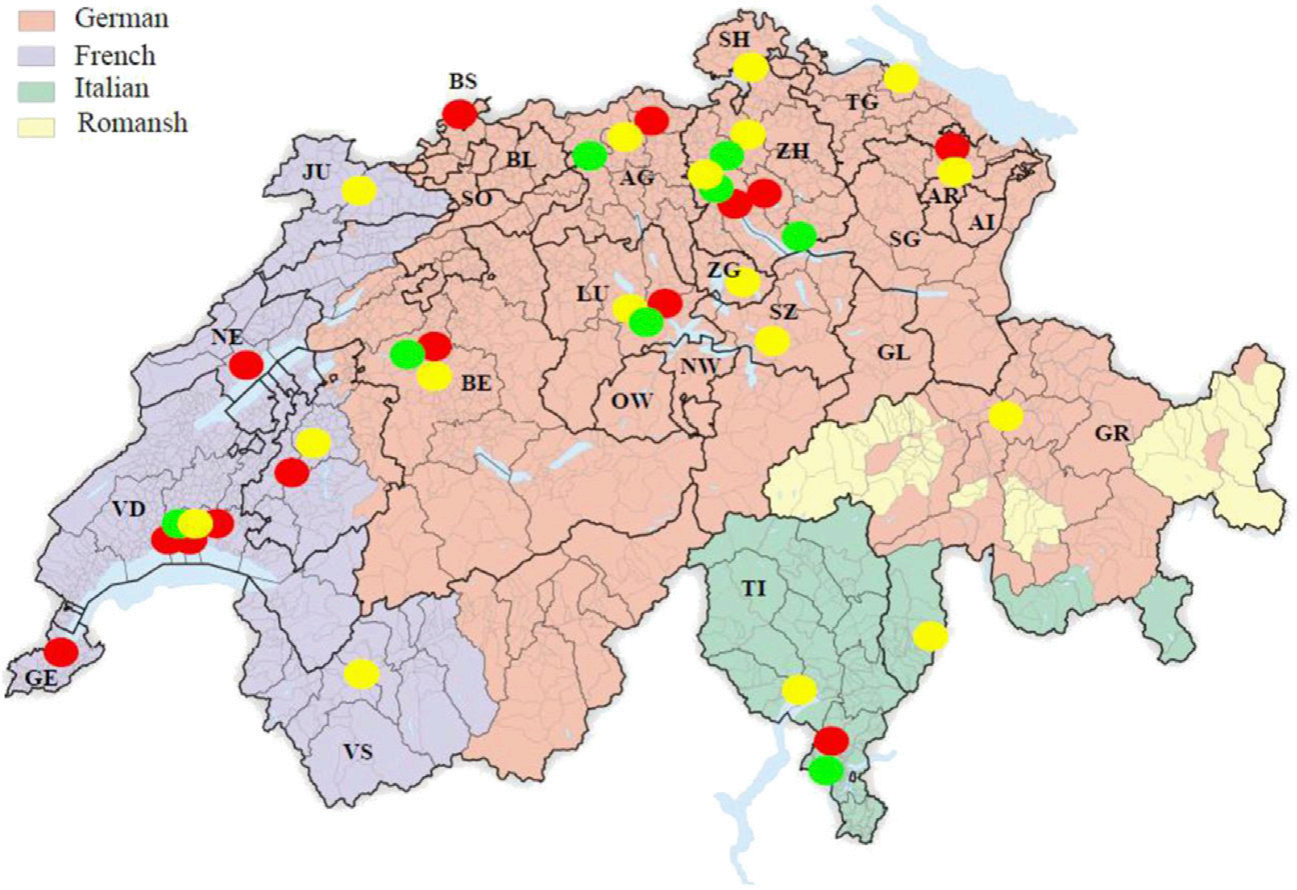
Map of Switzerland and location of Swiss higher education institutions, 2023 Cantons: GE-Geneva, VD-Vaud, VS-Valais, FR-Fribourg, NE-Neuchâtel, BE-Bern, JU-Jura, BS-Basel-Stadt, BL-Basel-Landschadt, SO-Solothurn, LU-Lucerne, OW-Obwalden, NW-Nidwalden, AG-Aargau, ZG-Zug, SZ-Schwyz, GL-Glarus, SH-Schauffhausen, ZH-Zürich, TG-Thurgau, AR-Appenzell Ausserhoden, AI-Appenzell Innerhoden, GR-Graubünden, TI-Ticino. Higher Education Institutions in red: The University of Basel (UniBas), the University of Bern (UniBe), the University of Fribourg (UniFr), the University of Geneva (UniGe), the University of Neuchâtel (UniNe), the University of Lausanne (UniL), the University of Lucerne (UniLu), the Università della Svizzera italiana (USI), the University of St. Gallen (UniSG), the École Polytechnique Fédérale de Lausanne (EPFL), the University of Zurich (UZH), the Swiss Federal Institute of Technology Zurich (ETH), the Paul Scherrer Institute (PSI), and Unisanté–Center for Primary Care and Public Health (Unisanté). Universities of teacher education are shown in yellow: the University of Teacher Education Vaud (VD), the University of Teacher Education Valais (VS), the University of Teacher Education Fribourg (FR), the University of Teacher Education Bern (BE), the University of Teacher Education BEJUNE (BEJUNE), the University of Teacher Education Lucerne (LU), the University of Teacher Education Zug (ZG), the University of Teacher Education Schwyz (SZ), the University of Teacher Education Northwestern Switzerland (PH FHNW), the University of Teacher Education Schaffhausen (SH), the Intercanton School for Special Needs Education (HfH), the University of Teacher Education Thurgau (TG), the University of Teacher Education St. Gallen (SG), the University of Teacher Education Graubünden (GR), and the Department of Education and Learning at the University of Applied Sciences and Arts of Southern Switzerland (SUPSI_DFA). Universities of applied sciences are shown in green: the University of Applied Sciences and Arts Western Switzerland (HES-SO), the Bern University of Applied Sciences (BFH), the University of Applied Sciences and Arts Northwestern Switzerland (FHNW), the Zurich University of Applied Sciences (ZFH), the Kalaidos University of Applied Sciences (Kalaidos-FH), the University of Applied Sciences of Eastern Switzerland (FHO), and the University of Applied Sciences and Arts of Southern Switzerland (SUPSI).

The sample of HEIs for which we have mapped OHS consists of 14 HEI including a majority of Lausanne’s HEIs, the cantonal universities of Berne, Basel, Geneva, Neuchâtel, Luzern, Lugano, Fribourg, Zürich, and St. Gallen, the Swiss Federal Institute of Technology Zürich (ETHZ), and the Paul Scherrer Institute (PSI) (Figure 1). Table 1 provides a short description and specific features of these HEIs.

**TABLE 1:** Swiss higher education institutions considered in the mapping study. Data source: Mapping study and inventory of Occupational Health Services in Swiss Higher Education Institutions, Switzerland, 2023.

Institution type, name, and location	Main language	Year of creation	Nb of students	Nb of PhD students	Nb of researchers staff	Nb of OHS^a^ provided
Universities						
University of Basel (UNIBAS)	German	1460	10,054	2946	5339	5
University of Berne (UNIBE)	German	1834	19,441	3,314	3,383	2
University of Fribourg (UNIFR)	French	1889	10,724	150	1441	4
University of Geneve (UNIGE)	French	1559	18,865	2325	4,837	6
University of Neuchatel (UNINE)	French	1838	4,508	619	533	2
University of Lausanne (UNIL)	French	1537	14,609	2532	2446	4
University of Lucerne (UNILU)	German	1600	2808	403	750	1
University of Lugano (USI)	Italian	1995	2923	477	907	1
University of St. Gallen (UNISG)	German	1898	8,694	597	548	3
University of Zurich (UZH)	German	1833	22,462	5659	6,557	5
Federal Institutes of Technology						
Federal Polytechnic School of Lausanne (EPFL)	French	1853	12,000	150	3,917	3
Federal Institute of Zurich (ETH)	German	1855	24,530	4,460	7,139	4
Hybrid institutions						
Paul-Scherrer-Institute (PSI), Villigen	German	1988	0	310	1364	3
Center for Primary Care and Public Health (UNISANTE), Lausanne	French	2019	0	20	100	4

^a^ OHS: Occupational Health Service. All data were collected in 2021. OHS, created after this date are not included in this table.

A wide variety of OHS have been identified. Each HEI has a different approach to OHS, but most offer help such as access to a general or occupational physician, psychological counselling/advice, and legal advice. Some HEIs offer additional services, as shown in Table 2. About 90% of the surveyed HEIs offer psychological support to researchers. This mainly consists of psychological counselling/support, usually limited to one or a few sessions, without offering a formal diagnosis or regular psychotherapy (for which referral to an external professional center may be provided). Information on how to access the OHS is generally well communicated, with clear and simple instructions for making an appointment. This information is available on the university website and is also disseminated through social media and university leaflets. Almost 70% of the HEIs provide OHS within the institution itself, while in the remaining HEIs, OHS are provided by an external specialized institution (e.g., Center for psychological support or psychotherapy).

**TABLE 2:** Type of occupational health services provided in the Swiss higher education institutions. Data source: Mapping study and inventory of Occupational Health Services in Swiss Higher Education Institutions, Switzerland, 2023.

Institutions	Occupational medicine consultation	Psychological counseling service	Juridical/legal counseling	Professional orientation counseling	Social affairs service	Administrative support	Spiritual counseling
UNIBAS	Y (c^a^, np^b^)	Y (b, 1)	Y (a, np)	Y (b, 1)	Y (b,1)	N	N
UNIBE	N	Y (a,1)	N	N	N	N	N
UNIFR	N	Y (a-b, 1–2)	N	N	N	Y (a, np)	Y (a, 1)
UNIGE	Y (a,1)	Y (a, 2)	N	N	N	N	N
UNINE	N	Y (b, 1)	N	N	Y (a, np)	N	N
EPFL	N	Y (b, 3)	N	N	Y (b, 1)	N	N
UNIL	Y (a, 1)	Y (b, 3)	N	N	Y (a, 1)	N	N
UNILU	N	Y (a, 1)	N	N	N	N	N
USI	N	Y (a, 1)	N	N	N	N	N
UNISG	N	Y (a, 1)	Y (a, 1)	N	N	Y (a,1)	N
UZH	Y (c, 1)	Y (a, 1)	N	N	N	N	N
ETH	N	Y (a, 1)	N	Y (c, np)	N	N	N
PSI	N	N	N	N	Y (c, 1)	Y (c, 1)	N
UNISANTE	Y (a,1)	Y (c, np)	NA	NA	Y (a, 1)	Y (a, 1)	N

Legend: Y, Yes, N, No.

^a^ Who this service is open/offered to: a-All b-Pre-graduated and PhD students c-Employees (e.g., researchers, professors, administrative and technical staff).

^b^ Conditions: 1- All consultations covered by the institution (free for user) but sometimes in limited number 2- Only the 1^st^ consultation is free, subsequent are charged at a reduced rate 3- Only the 1^st^ consultation is free, subsequent are charge at the normal rate (without discount) np-Not provided.

The review of available OHS showed that there are more services targeted at students than at research staff. Psychological counselling services are available to students in 13 out of 14 institutions, while only 9 institutions offer this service to research staff (Table 2). Services for students are also better advertised and promoted, whereas information on more general OHS is less visible. An important difference between HEIs in terms of price was observed for medical and especially psychological services. Some HEIs offer completely free consultations (e.g., covered by the university’s health insurance), while others offer only the first consultation free of charge, with subsequent consultations either covered by health insurance or remaining at the researcher’s expense. A few HEIs charge a relatively low fee starting from the first consultation.

To understand how institutional characteristics influence the availability of OHS in HEIs, we conducted a regression analysis examining the relationships between the number of students, PhD candidates, and research staff with the provision of various OHS. The analysis showed that the number of OHS provided by the HEIs is positively associated with the numbers of registered students and research staff employed by the corresponding HEIs [(β = 0.0001049, p = .03 for students; β = 0.0004148, p = .01 for research staff), Supplementary Table S1]. The association between the number of PhD students and the number of provided OHS was not statistically significant. In evaluating the OHS availability per type of service with a series of logistic regression analyses, a positive statistically significant association was observed between the availability of occupational medicine consultation and the number of research staff (β = 0.0003203, *p* = .01). The availability of juridical/legal counselling is associated with both the number of PhD students (β = 0.0001187, *p* = .02) and the number of research staff (β = 0.0000845, *p* = .04). Finally, the availability of professional orientation counselling is positively associated with the number of research staff (β = 0.0000923, *p* = .02).

### Qualitative Assessment of OSH Among Researchers

To fully test the protocol, the second sequence of the study was conducted as a pilot qualitative study assessing the researchers’ awareness of, and perceived utility of OHS provided within the Center of Primary Care and Public Health, University of Lausanne. This HEI includes about 100 researchers active in clinical research, health service research, public health, occupational and environmental health, and tropical medicine. We contacted 40 randomly selected professionals, representing professorial, intermediate, and early-career researchers (including PhD and post-doctoral fellows) and research support staff specializing in community health. Thirteen professionals expressed their interest to participate and twelve were interviewed (response rate 30%). The study sample description is summarized in Table 3.

**TABLE 3:** Description of participants in the pilot study conducted at one Swiss higher education institution. Study title: Qualitative Assessment of Occupational Health Services Among Researchers in Swiss Higher Education Institutions, Switzerland, 2023.

Characteristics	N	(%)
Gender		
Male	4	33.3
Female	8	66.7
Age		
40–59	5	41.6
20–39	7	58.4
Work position/function		
Research support person	3	25.0
Senior researchers	2	16.7
Post-doc	4	33.3
PhD student	3	25.0
Type of work contract		
Permanent	9	75.0
Temporary	3	25.0
Duration of work contract		
>4 years	5	41.6
3-4 years	5	41.6
<3 years	2	16.8
Total	12	100.0

The results of the thematic analysis (Table 4) indicate that the most commonly reported sources of stress were time pressure and work overload. To cope with stress, most researchers reported that they tried to take short mental breaks from work (e.g., exercise, talk to friends or a trusted colleague), but there is still a high demand for more institutional resources and a friendlier, more trusting, and helpful working environment. Notably, a quarter of the participants had experienced burnout, and most had sought and received help outside the institution. Among the four types of services available at this HEI, less than half of the participants could name them, and even fewer could give any details. Participants also expressed low interest and little confidence in existing OHS. Many perceived these services as non-anonymous and/or non-confidential, particularly in terms of hierarchical or managerial oversight, making them unlikely to seek help within the institution. The low level of awareness of OHS made it difficult to assess the usefulness or preventative effectiveness of these services. Participants’ suggestions for improving mental health support for researchers are presented in the Table 4.

**TABLE 4:** Thematic analysis of occupational health services at Swiss higher education institutions. Study title: Qualitative Assessment of Occupational Health Services Among Researchers in Swiss Higher Education Institutions, Switzerland, 2023.

Most stressful aspect of current job	Biggest drivers of burnout at workplace	Prevention ideas
• Time pressure• Work overload• Insufficient resources• Unclear expectations• Supervisor pressure• Lack of equality to grow	• Time pressure• Work overload• Insufficient resources• Communication problems• Chronic stress• Lack of job satisfaction• Lack of support• Interpersonal conflicts at work• Insufficient recognition• Lack of “balanced” life	• Helpline• Less time pressure• More support from supervisor/more mentoring for PhD• Anonymous and confidential solutions• Friendlier and warmer workspace• Recognition/appreciation• Hiring more people• More social events• More freedom to do things “my way”• Sport classes within work hours

## Discussion

In this study, we developed an original research protocol that sequentially combines a mapping study of OHS in a country- and HEI-specific manner with face-to-face semi-structured interviews with researchers. We tested the protocol and its components in Switzerland and confirmed its applicability in representing most HEI types within a country. Applied in the countries of the EU COST Action REMO and beyond, this protocol is likely to provide comprehensive and comparable data on the availability, accessibility, and usefulness of OHS in the HEIs. Despite the country-specific context and the inclusion of only one HEI in the thematic analysis, this study yielded several original outputs, namely the inventory of OHS in Swiss HEIs and insights into researchers’ awareness regarding these services.

The study results showed a diversity of OHS identified in Swiss HEIs, with important variations in the number, type, target groups, and financial accessibility of services across institutions. This heterogeneity points to possible inequalities in the approach to OHS across Swiss HEIs, despite the country’s small size. Some institutions, such as the University of Basel, provide a wide range of OHS, including occupational medicine consultations, psychological counselling, juridical/legal counselling, professional orientation counselling, and social affairs services. In contrast, others, such as the University of Bern, University of Lucerne, and the University of Lugano, offer only basic support limited to psychological counselling services. According to the European Agency for Security and Health at Work Report, institutions with effective practices should serve as models for others, promoting the sharing of best practices and standardizing service offerings to ensure uniform and effective mental health support across the academic community [29].

A quarter of researchers in our pilot study sample have already experienced mental health problems such as burnout, and few had turned to the services offered by their institution, preferring external solutions. This finding aligns with previous studies reporting that employees tend to find OHS difficult to access [30–32]. Notably, when asked why they preferred external services, participants cited confidentiality concerns as a key reason. Other potential factors, such as perceived quality or accessibility, were not raised by participants and thus not explored in this study. Future research should investigate these aspects to better understand preferences for external versus institutional mental health services. This corroborates our initial suspicion that researchers’ mental health issues may stem from inadequacies in OHS at their workplace, in terms of availability, accessibility, or effectiveness. Although we could not thoroughly explore the former two aspects in this pilot study, it appeared that the lack of researcher’s awareness of, and trust in, the OHS provided at their workplace is an important barrier to the preventive effectiveness of these services in addressing the specific needs of researchers.

The three most frequently cited sources of stress at work in this pilot qualitative study were time pressure, work overload and insufficient resources. The Early Career Researcher Survey conducted by the Swiss National Science Foundation (SNSF), using a large representative sample, revealed that besides the workload, early career researcher face problems with income and job security, which contribute to a stressful work environment, job dissatisfaction, and potentially worsen mental health concerns [33]. These findings underscore the need for academic institutions to enhance the preventive measures by providing support structures and OHS, aligning with broader challenges identified by the SNSF survey. A recent *Nature* report cited some examples of such support, namely the mental health awareness courses and workshops launched by the University of Zurich and Imperial College London [34].

### Study Limitations and Strengths

These initial findings are based on a small sample size within a small and atypical HEI, where researchers make up only 10% of the workforce. This calls for cautious interpretation and limits the generalizability of the results. Conversely, this can be an advantage for the protocol testing before its application in other HEIs. To draw definitive conclusions, the study should be conducted in multiple HEIs of varying sizes and profiles, and in different countries.

The HEI selected for this pilot study presented constraints in the availability and scope of its OHS, potentially limiting the diversity of services captured. Additionally, there is a risk of self-selection bias in the qualitative phase, as individuals experiencing mental health issues may have been either more or less likely to participate, potentially skewing the results [35]. Yet, the 30% response rate is higher than rates usually observed in studies based on online invitations [36, 37] but lower than those invited by an occupational physician [38, 39]. On the other side, the sensitive nature of mental health issues in academia and/or the worries regarding data confidentiality, as indicated by the refusal of interview recording by all 12 participants, can be additional features, potentially affecting data quality. The exact mechanisms could be investigated in a larger multicentric study applying this protocol. Finally, given the limited awareness regarding provided OHS and their use by the study participants, we could not adhere to a formal evaluative framework within this pilot study. However, the study design allows extending the protocol to integrate specific theoretical models such as the Service needs coverage model [40] to evaluate the extent to which existing resources in the HEI effectively meet the needs of the researchers. This model is useful for identifying gaps in service provision and strategies to improve coverage and accessibility of the services from the users’ perspective [41]. Alternatively, the Model of access to care allows for the evaluation of the availability, accessibility, accommodation, affordability and accessibility of the healthcare for users (i.e., researchers) [42].

The study has several strengths. First, the integration of qualitative and quantitative approaches, as outlined by Onwuegbuzie and Leech [43], provides researchers with a multifaceted lens trough which to analyze and interpret data. This combination allows for a comprehensive exploration of complex phenomena, using the numerical precision and statistical rigor of quantitative data alongside the rich context and nuanced understanding provided by qualitative data. By synthesizing these methods, researchers gain deeper insights into their research question, leading to improved interpretation of findings. Furthermore, the study’s use of a multi-level sequential design, as highlighted by Leech and Onwuegbuzie [44], allows for a systematic and comprehensive exploration of the research topic by integrating both quantitative and qualitative perspectives. This design facilitates analytical generalizations and the transfer of findings across cases, thereby enhancing the applicability and relevance of the study. In addition, the sequential-multilevel sampling design used in the study, as defined by [45], incorporates two crucial aspects: the temporal orientation of the components and the relationship between the two samples. The sequential aspect ensures that the quantitative and qualitative phases take place consecutively, with the inventory and systematic review preceding the semi-structured interviews. As for the multi-level aspect, it involves the uses of samples drawn from different levels of population of interest, such as senior researchers and PhD students, thus providing a comprehensive understanding of the research topic. Overall, these methodological strengths enhance the reliability and validity of the harmonized study protocol, allowing for a nuanced exploration of the wide variation in OHS provision and awareness thereof among academics.

### Conclusion

This two-part study has made it possible, on the one hand, to take stock of OHS in Swiss HEIs and, on the other hand, to analyze the level of effectiveness and awareness of researchers regarding these OHS. Using a comprehensive protocol combining both qualitative and quantitative methods, this study demonstrated the crucial role that OHS play in the health, particularly the mental health, of academic staff. The results of this research highlighted significant differences in the provision of OHS across different HEIs, with implications for researchers’ access to and use of these services. The analysis highlighted barriers related to accessibility and awareness, with academic staff preferring to use services outside the institution when experiencing mental health problems. Study participants reported a lack of awareness of available OHS aimed at protecting and promoting their mental health. In addition to their limited knowledge of the services, they also expressed a reluctance to use them because of concerns pertaining to anonymity and confidentiality. As a result, there is a real lack of interest and trust in these services, which may exacerbate the difficulties identified by HEI staff. To gain a fuller understanding and to inform targeted interventions, this study needs to be extended to a wider range of countries and HEIs, including institutions of varying size, regions, and academic specialization. Given the significant differences observed between institutions, future efforts should aim to establish standardized guidelines and frameworks for OHS provision. This would help ensure equitable, high-quality support services across HEIs, enabling all researchers, regardless of their institution, to access appropriate resources for their mental health and wellbeing. In addition, replicating this study in other national and regional contexts would be valuable to assess the applicability and relevance of the study protocol and to explore how local conditions may shape OHS provision in academia. Ultimately, this study highlights the need for a dialogue and action to prioritize mental health in HEIs. By addressing the identified gaps in OHS provision and promoting greater awareness and accessibility, we can strive to create environments where researchers feel supported, valued, and empowered to thrive in their academic pursuits.
