# Supplementary material for: Inventory of Mental Health Services in Academia and Researchers’ Awareness of Their Availability: Mixed Method Research Protocol and Pilot Study in Switzerland
Source: Int J Public Health. 2025 May 21;70:1607982. doi: 10.3389/ijph.2025.1607982 (PMC12133474; doi:10.3389/ijph.2025.1607982)
Supplement: Supplementary file 1 [file Table1.DOCX]

**Table S1: Univariable and multivariable regression of occupational health services and number of students, PhD, research staff**

| **Independent**  **variable** | **Univariable analysis** | | | **Multivariable analysis** | | | |
| --- | --- | --- | --- | --- | --- | --- | --- |
|  | **Coefficient** | **95% CI** | **P value** | **Adjusted variables** | **Coefficient** | **95% CI** | **P value** |
| **Number of Occupational services (Linear regression)** | | | | | | | |
| No. of students | 0.0001049 | (0.0000124, 0.0001974) | 0.03* | No. of PhD, No. of research staff | 0.0000257 | (-0.0001719, 0.0002233) | 0.78 |
| No. of PhD | 0.0004304 | (-0.0000035, 0.0008643) | 0.05 | No. of students, No. of research staff | -0.0001727 | (-0.0010765, 0.0007312) | 0.68 |
| No. of research_staff | 0.0004148 | (0.0001228, 0.0007068) | 0.01* | No. of students, No. of PhD | 0.0004527 | (-0.0002514, 0.0011568) | 0.18 |
| **Availability of the Occupational medicine consultation (Logistic regression)** | | | | | | | |
| No. of students | -0.0000118 | (-0.000049, 0.0000254) | 0.50 | No. of PhD, No. of research staff | -0.0000462 | (-0.0001063, 0.0000139) | 0.12 |
| No. of PhD | -0.0000555 | (-0.0002226, 0.0001115) | 0.48 | No. of students, No. of research staff | -0.0002378 | (-0.0005127, 0.0000372) | 0.08 |
| No. of research_staff | 0.0000294 | (-0.0001, 0.0001587) | 0.63 | No. of students, No. of PhD | 0.0003203 | (0.0001061, 0.0005345) | 0.01* |
| **Availability of the Psychological counseling service (Logistic regression)** | | | | | | | |
| No. of students | 0.0000123 | (-0.0000066, 0.0000311) | 0.18 | No. of PhD, No. of research staff | 0.0000153 | (-0.0000288, 0.0000594) | 0.46 |
| No. of PhD | 0.0000388 | (-0.0000497, 0.0001272) | 0.36 | No. of students, No. of research staff | -0.0000369 | (-0.0002384, 0.0001647) | 0.69 |
| No. of research_staff | 0.0000363 | (-0.0000301, 0.0001028) | 0.26 | No. of students, No. of PhD | 0.0000156 | (-0.0001414, 0.0001726) | 0.83 |
| **Availability of the Juridical/legal counseling (Logistic regression)** | | | | | | | |
| No. of students | 0.0000123 | (-0.0000143, 0.0000389) | 0.33 | No. of PhD, No. of research staff | -0.0000482 | (-0.0000875, -8.96e-06) | 0.02* |
| No. of PhD | 0.0001187 | (0.0000189, 0.0002185) | 0.02* | No. of students, No. of research staff | 0.0001856 | (5.99e-06, 0.0003651) | 0.04* |
| No. of research_staff | 0.0000845 | (5.16e-06, 0.0001638) | 0.04* | No. of students, No. of PhD | 0.0001042 | (-0.0000357, 0.0002441) | 0.13 |
| **Availability of the Professional orientation counseling (Logistic regression)** | | | | | | | |
| No. of students | 0.0000146 | (-0.0000115, 0.0000407) | 0.25 | No. of PhD, No. of research staff | -0.0000309 | (-0.0000773, 0.0000155) | 0.17 |
| No. of PhD | 0.0000912 | (-0.0000194, 0.0002019) | 0.09 | No. of students, No. of research staff | 0.0000168 | (-0.0001953, 0.0002289) | 0.86 |
| No. of research_staff | 0.0000923 | (0.0000165, 0.000168) | 0.02* | No. of students, No. of PhD | 0.0001717 | (6.48e-06, 0.000337) | 0.04* |
| **Availability of the Social affairs service (Logistic regression)** | | | | | | | |
| No. of students | -0.0000238 | (-0.00006, 0.0000125) | 0.18 | No. of PhD, No. of research staff | -0.0000389 | (-0.0001226, 0.0000448) | 0.32 |
| No. of PhD | -0.0000825 | (-0.0002509, 0.000086) | 0.31 | No. of students, No. of research staff | -0.0000215 | (-0.0004045, 0.0003614) | 0.90 |
| No. of research_staff | -0.0000503 | (-0.0001815, 0.000081) | 0.42 | No. of students, No. of PhD | 0.0000778 | (-0.0002205, 0.0003761) | 0.57 |
| **Availability of the Administrative support (Logistic regression)** | | | | | | | |
| No. of students | 2.11E-06 | (-0.0000336, 0.0000378) | 0.90 | No. of PhD, No. of research staff | 0.0000113 | (-0.0000699, 0.0000925) | 0.76 |
| No. of PhD | -0.0000199 | (-0.0001803, 0.0001406) | 0.79 | No. of students, No. of research staff | -0.0001407 | (-0.0005121, 0.0002308) | 0.42 |
| No. of research_staff | 0.0000107 | (-0.0001123, 0.0001337) | 0.85 | No. of students, No. of PhD | 0.0000697 | (-0.0002197, 0.0003591) | 0.61 |
| **Availability of a Spiritual counseling (Logistic regression)** | | | | | | | |
| No. of students | -1.20E-07 | (-0.0000205, 0.0000203) | 0.99 | No. of PhD, No. of research staff | 0.000026 | (-0.0000166, 0.0000686) | 0.20 |
| No. of PhD | -0.0000358 | (-0.0001247, 0.0000532) | 0.40 | No. of students, No. of research staff | -0.0001081 | (-0.0003028, 0.0000866) | 0.24 |
| No. of research_staff | -0.0000183 | (-0.0000876, 0.000051) | 0.57 | No. of students, No. of PhD | -0.0000237 | (-0.0001753, 0.000128) | 0.74 |

*p value 0.05
